# Identification of Compounds with Anti-Proliferative Activity against *Trypanosoma brucei brucei* Strain 427 by a Whole Cell Viability Based HTS Campaign

**DOI:** 10.1371/journal.pntd.0001896

**Published:** 2012-11-29

**Authors:** Melissa L. Sykes, Jonathan B. Baell, Marcel Kaiser, Eric Chatelain, Sarah R. Moawad, Danny Ganame, Jean-Robert Ioset, Vicky M. Avery

**Affiliations:** 1 Discovery Biology, Eskitis Institute for Cell and Molecular Therapies, Griffith University, Nathan, Queensland, Australia; 2 The Walter and Eliza Hall Institute of Medical Research, Parkville, Victoria, Australia; 3 Department of Medical Biology, The University of Melbourne, Parkville, Victoria, Australia; 4 Department of Medicinal Chemistry, Faculty of Pharmacy and Pharmaceutical Sciences, Monash Institute of Pharmaceutical Sciences, Monash University, Parkville, Victoria, Australia; 5 Swiss Tropical and Public Health Institute, Basel, Switzerland; 6 University of Basel, Basel, Switzerland; 7 Drugs for Neglected Diseases initiative (DNDi), Geneva, Switzerland; Universidad Autónoma de Yucatán, Mexico

## Abstract

Human African Trypanosomiasis (HAT) is caused by two trypanosome sub-species, *Trypanosoma brucei rhodesiense* and *Trypanosoma brucei gambiense*. Drugs available for the treatment of HAT have significant issues related to difficult administration regimes and limited efficacy across species and disease stages. Hence, there is considerable need to find new alternative and less toxic drugs. An approach to identify starting points for new drug candidates is high throughput screening (HTS) of large compound library collections. We describe the application of an Alamar Blue based, 384-well HTS assay to screen a library of 87,296 compounds against the related trypanosome subspecies, *Trypanosoma brucei brucei* bloodstream form lister 427. Primary hits identified against *T.b. brucei* were retested and the IC_50_ value compounds were estimated for *T.b. brucei* and a mammalian cell line HEK293, to determine a selectivity index for each compound. The screening campaign identified 205 compounds with greater than 10 times selectivity against *T.b. brucei*. Cluster analysis of these compounds, taking into account chemical and structural properties required for drug-like compounds, afforded a panel of eight compounds for further biological analysis. These compounds had IC_50_ values ranging from 0.22 µM to 4 µM with associated selectivity indices ranging from 19 to greater than 345. Further testing against *T.b. rhodesiense* led to the selection of 6 compounds from 5 new chemical classes with activity against the causative species of HAT, which can be considered potential candidates for HAT early drug discovery. Structure activity relationship (SAR) mining revealed components of those hit compound structures that may be important for biological activity. Four of these compounds have undergone further testing to 1) determine whether they are cidal or static *in vitro* at the minimum inhibitory concentration (MIC), and 2) estimate the time to kill.

## Author Summary

Human African Sleeping Sickness (HAT) is a disease caused by sub-species of *Trypanosoma*. The disease affects developing countries within Africa, mainly occurring in rural regions that lack resources to purchase drugs for treatment. Drugs that are currently available have significant side effects, and treatment regimes are lengthy and not always transferrable to the field. In consideration of these factors, new drugs are urgently needed for the treatment of HAT. To discover compounds suitable for drug discovery, cultured trypanosomes can be tested against libraries of compounds to identify candidates for further biological analysis. We have utilised a 384-well format, Alamar Blue viability assay to screen a large non-proprietary compound collection against *Trypanosoma brucei brucei* bloodstream form lister 427. The assay was shown to be reproducible, with reference compounds exhibiting activity in agreement with previously published results. Primary screening hits were retested against *T.b. brucei* and HEK293 mammalian cells in order to assess selectivity against the parasite. Selective hits were characterised by chemical analysis, taking into consideration drug-like properties amenable to further progression. Priority compounds were tested against a panel of protozoan parasites, including *Trypanosoma brucei rhodesiense*, *Trypanosoma cruzi*, *Leishmania donovani* and *Plasmodium falciparum*. Five new compound classes were discovered that are amenable to progression in the drug discovery process for HAT.

## Introduction

Human African Trypanosomiasis (HAT) is caused by infection with either the trypanosome subspecies *Trypanosoma brucei gambiense* or *Trypanosoma brucei rhodesiense*. Decreasing numbers of reported new cases over the last 10 years have been reported - from over 25,000 in 2000 to 10,000 in 2009 - of which over 95% are caused by *T.b. gambiense* [1]. However, the World Health Organization (WHO) currently estimates the actual number of cases to be around 30,000 [http://www.who.int/mediacentre/factsheets/fs259/en/]. HAT is mainly confined within sub-Saharan Africa, where the vector, the parasite and the animal reservoirs co-exist [2]. HAT occurs in two stages, whereby the first stage, also called the haemolymphatic stage, corresponds to the invasion of lymph, blood and other tissues by the trypanosomes, and the second stage is associated with parasites crossing the blood-brain barrier and invading the central nervous system (CNS). Symptoms of the second stage of the disease include mental impairment, severe headaches, fever, chronic encephalopathy and an eventual, terminal somnolent state, if the disease remains untreated.

There are currently few drugs available for the treatment of HAT. For the first stage of the disease, suramin is used as the treatment for *T.b. rhodesiense* and pentamidine for *T.b. gambiense* infections. Neither of these drugs are able to cross the blood brain barrier and therefore are not effective against the CNS resident, second stage of the disease. In addition, both of these treatments have significant side effects, often resulting in reduced compliance. Suramin is associated with exfoliative dermatitis [2] and renal failure [3], whilst pentamidine use has been correlated with diabetes mellitus and nephrotoxicity [4]. Melarsoprol, an organoarsenic compound, is most frequently used for the treatment of the second stage of the disease as it is effective against both trypanosome subspecies. However, there have been reports of high failure rates with melarsoprol, and although resistance has not definitively been proven, this does highlight the need for alternative therapies [5]. As a consequence of treatment with melarsoprol, encephalopathic syndromes occur in 5 to 10% of all of treated patients causing between 10 to 70% fatality, depending on the literature source [6]–[10]. The alternative therapy for the second stage of the disease, eflornithine, is a less toxic and a safer alternative however it is unfortunately not effective against *T.b. rhodesiense*. There are also problems with affordability of eflornithine in many of the disease-endemic countries [11]. The recent inclusion of nifurtimox to the WHO Essential Medicine List in 2009 [11], [12], to be used only in combination with eflornithine for the treatment for the second stage of HAT caused by *T.b. gambiense*, is a significant milestone. Nifurtimox-eflornithine combination therapy (NECT) has a shorter and simplified administration regimen and is the only significantly improved treatment option made available to patients in the past 25 years. NECT is now used as the first line treatment for stage 2 HAT caused by *T.b. gambiense* [13], [14]. There was some hope for an oral drug for treating the first stage of HAT with the compound, pafuramidine (DB289). Unfortunately, in an extended phase III trial, liver toxicity and delayed renal insufficiency were observed in a number of patients and consequently the program was discontinued in 2008 [15]. Recent advances which hold promise include the identification of orally bioavailable oxaborole 6-carboxamides which have been shown to cure a murine model of late stage CNS HAT [16] and an orally active benzoxaborole has been selected to enter pre-clinical studies [17]. Despite this there is still a need for the discovery of additional trypanocidal compounds with the potential for further progression in the drug discovery pipeline for HAT. This is particularly evident when one takes into account the toxicity of traditional treatments, the inability of the newer less toxic combination therapies to treat both subspecies or both disease stages, and the historical 90% failure rate of drugs entering the clinic to reach the market [18].

One method for the identification of active compounds against HAT is the application of high throughput screening (HTS) methods. HTS against *T.b. brucei* targets, such as the enzyme TbHK1 (*Trypanosoma brucei* hexokinase 1) [19] have recently been reported. A potential drawback to target-based HTS is that screening hits may have to undergo significant medicinal chemistry optimisation to impart favourable properties for low serum binding, high membrane permeability and high aqueous solubility in order to register potent activity against the parasite. Whole cell screening is becoming increasingly popular, as although elucidation of the biological target requires deconvolution, active compounds are discovered under conditions that are already physiologically relevant. We have recently reported the development of a 384 Alamar Blue based 384-well viability assay for HTS screening of compounds against *T.b. brucei* [20]. For this assay, and indeed many *in vitro* models for studies of HAT, the human non-infective sub-species *T.b. brucei* blood stream form has been utilised [21]. Alamar Blue (containing resazurin) is a fluorometric/colorimetric REDOX indicator. In a reducing environment caused by metabolising cells, resazurin is converted to resorufin, a fluorescent end product. This reagent has been used routinely as an indicator of the viability of mammalian cells. It is thought that cells may induce a reduction in the medium or reduce Alamar Blue intracellularly [22]. We have shown that the fluorescent Alamar Blue signal is linear to the number of *T.b. brucei* cells in a well, therefore it provides a good indication of viable cell numbers [20]. For this reason we have used this assay to assess the activity of compounds against *T.b. brucei* whole cells.

Here we describe the HTS of a compound library (WEHI 2003 collection [23]) using a 384-well whole cell *T.b. brucei* assay, and the retesting of the identified active compounds against both *T.b. brucei* and a human cell line, HEK293, in order to assess mammalian cytotoxicity. The reproducibility of both the primary and retest assays were evaluated by the Z'-factor (Z'), a coefficient which reflects the reproducibility of the assay and is calculated using the positive and negative controls. The Z' takes into account the control signal range and variation, with a value close to 1 considered highly reproducible [24]. Reference compound activities for the *T.b. brucei* assay were compared with previously published results for the same assay format [20], [25]. Selectively active compounds were subjected to rigorous chemical analysis taking into account drug like and non-drug like structural properties. The selectivity index (SI) was defined as the HEK293 IC_50_ values divided by the *T.b. brucei* IC_50_ value. The compounds selected, with the initial criteria of an SI of greater than 10 times, were ultimately shown to have SI values ranging from 19 and a predicted value greater than 345. Further testing against *T.b. rhodesiense* revealed five new classes of active compounds that are recommended as chemical leads for the potential development of therapeutics against HAT. SAR mining revealed components of these hit compound structures that may be important for the observed biological activity, and these will be outlined. Based on compound availability, four compounds were selected for further biological profiling by estimating the time to kill and assessment if the compound action is cidal.

## Methods

### *In vitro* culture of *T.b. brucei* and HEK293 cells

*T.b. brucei* lister 427 cells [26] were maintained in log phase growth in 25 cm^2^ tissue culture flasks (Corning, NY, USA) by sub-culturing at either 24 or 48 hour intervals. Cells were grown in HMI-9 medium [27], supplemented with 10% fetal calf serum (FCS) and 100 IU/ml penicillin/streptomycin (Invitrogen, Carlsbad, California, USA) with incubation at 5% CO_2_ at 37°C in humidified conditions. HEK293 cells were maintained in high glucose DMEM with L-glutamine, supplemented with 1× non-essential amino acids (NEAA; Invitrogen, USA) and 1 mM sodium pyruvate. Growth conditions were in 5% CO_2_ at 37°C, under humidified conditions.

### *T.b. brucei* Alamar Blue viability estimation assay

All reagent and cell additions were made with a Multidrop liquid handler (Thermo Scientific, Newington, NH, USA) under sterile conditions. Fifty-five microliters of 2000 cells/mL of *T.b. brucei* in HMI-9 medium were added to a black, clear-bottomed 384-well lidded plate (BD Biosciences, Franklin Lanes, NJ, USA). Cells were incubated for 24 hours at 37°C in an atmosphere of 5% CO_2_ before addition of 5 µl of compounds/DMSO for control wells. Compounds suspended in 100% DMSO or 100% DMSO as controls were pre-diluted 1∶21 in high glucose DMEM without FCS by using a Minitrack robotic liquid handler (PerkinElmer, Waltham, MA, USA). Five microliters of diluted sample was added to the plate to give a final DMSO concentration of 0.417% in the assay. Cells were incubated for an additional 48 hours at 37°C. Ten microliters of 70% Alamar Blue (Biosource, Bethesda, MD, USA) was added to each well (diluted in HMI-9 medium supplemented with 10% FCS) to a final concentration of 10% in the assay. The plate was incubated for two hours under the same conditions, then incubated for 22 hours in the dark at room temperature. Wells were read at 535 nm (excitation) and 590 nm (emission) wavelengths on a Victor II Wallac plate reader (PerkinElmer, USA). Specific dilutions are explained further in the primary and retest assay methodology. Reference drugs used in the assay were pentamidine (Sigma-Aldrich, St Louis, MO, USA), diminazene aceturate (Sigma-Aldrich, USA) and puromycin (Calbiochem, San Deigo, CA, USA). Pentamidine is used to treat patients with HAT and diminazene is a veterinary drug used against *T.b. brucei* to combat infections in cattle. Puromycin is a non selective, protein synthesis inhibitor.

### HEK293 Alamar Blue viability estimation assay

Cells at 80% confluence were harvested and diluted in growth medium (high glucose DMEM supplemented with 10% FCS) to 7.27×10^4^ cells/ml. Under sterile conditions, 55 µl of diluted cells per well were added to a black, clear bottomed 384- well lidded plate (BD Biosciences, Bedford, MA, USA) with a Multidrop liquid handler (Thermo Scientific, Barrington, IL, USA). Incubation times, compound additions and plate read were as per the trypanosome viability assay, with the exception that Alamar Blue was diluted in HEK293 growth media before addition, and incubation of Alamar Blue at 37°C, in 5% CO_2_, was for 4 hours, followed by incubation at room temperature for 20 hours. The activity of compounds against HEK293 cells was used to calculate the SI of mammalian to *T.b. brucei* cells. The control compound for HEK293 cells was puromycin (Calbiochem, USA).

### L6 viability estimation assay

L6 rat skeletal myoblasts [28], [29] were purchased from the American Type Culture Collection (ATCC, Rockville, MD, USA; ATCC number CRL 1458). This cell line was used for cytotoxicity testing to calculate an SI against *T.b. rhodesiense* and screened alongside the *T.b. rhodesiense*, *P. falciparum*, *T. cruzi* and *L. donovani* assays. L6 were also the host cells for the *T. cruzi* assay. Assays were performed in 96-well microtiter plates, each well containing 100 µl of RPMI 1640 medium supplemented with 1% L-glutamine (200 mM), 10% FCS, and 4000 L6 cells. Serial drug dilutions of eleven 3-fold dilution steps covering a range from 100 to 0.002 µg/ml were prepared. After 70 hours of incubation the plates were inspected under an inverted microscope to assure growth of the controls and sterile conditions. Ten µl of resazurin solution (resazurin, 12.5 mg in 100 ml double-distilled water) was then added to each well and the plates incubated for another 2 hours. Then the plates were read with a Spectramax Gemini XS microplate fluorometer (Molecular Devices Cooperation, Sunnyvale, CA, USA) using an excitation wavelength of 536 nm and an emission wavelength of 588 nm. Data was analysed using the microplate reader software Softmax Pro (Molecular Devices, USA). Podophyllotoxin was used as a positive control in the assay.

### *T. b. rhodesiense* STIB900 assay

*T.b. rhodesiense* STIB900 stock was isolated in 1982 from a human patient in Tanzania and after several mouse passages cloned and adapted to axenic culture conditions [30]. Fifty microliters of Minimum Essential Medium (MEM) supplemented with 25 mM HEPES, 1 g/l additional glucose, 1% MEM non-essential amino acids (100×), 0.2 mM 2-mercaptoethanol, 1 mM Na-pyruvate and 15% heat inactivated horse serum was added to each well of a 96-well microtiter plate. Serial drug dilutions of eleven 3-fold dilution steps covering a range from 100 to 0.002 µg/ml were prepared. Four thousand bloodstream form cells of *T.b. rhodesiense* STIB 900 in 50 µl were added to each well and the plate incubated at 37°C under a 5% CO_2_ atmosphere for 70 hours. Ten microlitres of resazurin solution (resazurin, 12.5 mg in 100 ml double-distilled water) was then added to each well and incubation continued for a further 2–4 hours [31]. Plates were then read with a Spectramax Gemini XS microplate fluorometer (Molecular Devices, USA) using an excitation wavelength of 536 nm and an emission wavelength of 588 nm. Data was analysed using the microplate reader software Softmax Pro (Molecular Devices, USA). The drug melarsoprol was a positive control against *T.b. rhodesiense*.

### *T. cruzi* Tulahuen strain C2C4 β-galactosidase assay

Rat skeletal myoblasts (L6 cells) were seeded in 96-well microtitre plates at 2000 cells/well in 100 µl RPMI 1640 medium with 10% FCS and 2 mM l-glutamine. After 24 hours the medium was removed and replaced by 100 µl per well containing 5000 trypomastigote forms of *T. cruzi* Tulahuen strain C2C4 containing the β-galactosidase (Lac Z) gene [32]. After 48 hours the medium was removed from the wells and replaced by 100 µl fresh medium with or without a serial drug dilution of eleven 3-fold dilution steps covering a range from 100 to 0.002 µg/ml. After 96 hours of incubation the plates were inspected under an inverted microscope to assure growth of the controls and sterility. Then 50 µl of the substrate, containing chlorophenol red-β-D-galactopyranoside (CPRG) and Nonidet, was added to all wells. A colour reaction developed within 2–6 hours that could be read photometrically at 540 nm. Data were transferred into the graphic programme Softmax Pro (Molecular Devices, USA), which calculated IC_50_ values. The drug benznidazole was used as a positive standard in this assay.

### *L. donovani* axenic amastigote fluorescence assay

Amastigotes of *L. donovani* strain MHOM/ET/67/L82 were grown in axenic culture at 37°C in SM medium [33] at pH 5.4 supplemented with 10% heat-inactivated FCS under an atmosphere of 5% CO_2_ in air. One hundred µl of culture medium containing 10^5^ amastigotes from axenic culture with or without a serial drug dilution were seeded in 96-well microtitre plates. Serial drug dilutions of eleven 3-fold dilution steps covering a range from 100 to 0.002 µg/ml were prepared. After 70 hours of incubation the plates were inspected under an inverted microscope to assure growth of the controls and sterile conditions. Ten µl of resazurin solution (12.5 mg resazurin dissolved in 100 ml distilled water) [34] were then added to each well and the plates incubated for another 2 hours. The plates were then read with a Spectramax Gemini XS microplate fluorometer (Molecular Devices, USA) using an excitation wavelength of 536 nm and an emission wavelength of 588 nm. Data was analysed using the software Softmax Pro (Molecular Devices, USA). Decrease of fluorescence ( = inhibition) was expressed as percentage of the fluorescence of control cultures and plotted against the drug concentrations. From the sigmoidal inhibition curves the IC_50_ values were calculated. Miltefosine served as a known drug control in this assay.

### *P. falciparum* ^3^Hypoxanthine assay

*In vitro* activity against erythrocytic stages of *P. falciparum* was determined using a ^3^H-hypoxanthine incorporation assay [35], [36] using the chloroquine and pyrimethamine resistant K1 strain that originates from Thailand [37]. Compounds dissolved in DMSO at 10 mg/ml were added to parasite cultures incubated in RPMI 1640 medium without hypoxanthine, supplemented with HEPES (5.94 g/l), NaHCO_3_ (2.1 g/l), neomycin (100 U/ml), Albumax (5 g/l) and washed human A^+^ red blood cells at 2.5% haematocrit (0.3% parasitaemia). Serial drug dilutions of eleven 3-fold dilution steps covering a range from 100 to 0.002 µg/ml were prepared. The 96-well plates were incubated in a humidified atmosphere at 37°C; 4% CO_2_, 3% O_2_, 93% N_2_. After 48 hours, 50 µl of ^3^H-hypoxanthine ( = 0.5 µCi) was added to each well of the plate. The plates were incubated for a further 24 hours under the same conditions. The plates were then harvested with a Betaplate cell harvester (Wallac, Zurich, Switzerland), and the red blood cells transferred onto a glass fibre filter then washed with distilled water. The dried filters were inserted into a plastic foil with 10 ml of scintillation fluid, and counted in a Betaplate liquid scintillation counter (Wallac, Zurich, Switzerland). IC_50_ values were calculated from sigmoidal inhibition curves using Microsoft Excel. Chloroquine was used as a positive control in the hypoxanthine assay.

### Primary screening campaign

Primary screening of the library, consisting of 87,296 compounds in two hundred and forty eight 384-well plates, was undertaken in single point against *T.b. brucei*. Stock solutions consisted of test compound at a concentration of 5 mM in 100% DMSO. One µl of each compound stock solution was diluted by the addition of 40 µl of dilution medium (high glucose DMEM without FCS) by a multidrop liquid handler (Thermo Scientific, USA). A 5 µl sample of this diluted solution was then added to the trypanosome assay plate. The final concentration of test compound in the assay was 10.2 µM and that of DMSO was 0.42% v/v. Compounds were screened over a total of 11 days, at an average of 80 plates per day, taking into consideration that the assay incubation was 3 days total. Test compounds were added to plates in batches of 20 at two hour intervals, to maintain the timing of additions and reads.

Compound activity was calculated as the percentage inhibition in relation to positive and negative controls. The positive control, pentamidine, was contained in whole control plates, separate to the plates containing compounds, and the negative control (no effect) comprised of 0.42% DMSO, in column 24 of each test compound assay plate. These in-plate negative controls were used in an effort to normalise compound activity in relation to any plate to plate variation in the assay signal. A whole 384-well control plate was included in each day's screening, one per 20 compound plates containing half a plate of 2 µM pentamidine for the positive assay control, and half a plate of 0.42% v/v DMSO as a negative control. The positive assay control was used to calculate compound activity for batches of 20 compound plates. As well providing the positive control data, these whole plate controls were used for the calculation of the Z' to measure the reproducibility of the assay. An active hit was defined as a compound that demonstrated greater than the mean percentage activity of the library, plus three times the standard deviation. A separate plate containing a 13 point dose-response of reference compounds in triplicate was also included per 20 test plates to calculate the sensitivity of the assay.

### Retest library screening hits

Compounds identified from primary screening were retested against both *T.b. brucei* and HEK293 cells in duplicate and at varying concentrations to obtain a dose-response curve. Thus, a 5 µl sample of fresh compound stock solution (5 mM in DMSO) was cherry picked into 384-well plates and diluted 1∶10 in dilution medium (high glucose DMEM without FCS). Serial dilutions of these samples were then prepared in the same media by a Minitrak robotic liquid handler (Perkin Elmer, USA). This resulted in a total 13 doses per sample with 41.7 µM as the highest concentration of test compound, for which the DMSO concentration was 0.83% v/v. A screening dose of 10.4 µM was included in the dilution series to enable reconfirmation of primary screening *T.b. brucei* activity. Compounds with activity against *T.b. brucei* ≤10 µM, which also displayed an SI of ≥10, were selected for medicinal chemistry analysis.

The DMSO working concentration in the serial dilutions was maintained at 5%, giving a final assay concentration of 0.42% DMSO, except for the 41.7 µM test compound solution, where as previously stated the corresponding DMSO concentration was 0.83% v/v. The concentration of DMSO that can be tolerated in the *T.b. brucei* assay has been previously determined as 0.42% [20]. Therefore the 41.7 µM test compound sample with 0.83% v/v DMSO was not used in the *T.b. brucei* assay and thus the top test compound concentration in this assay was 20.8 µM. However, as the HEK293 assay can tolerate 0.83% DMSO (results not shown) the highest test compound concentration of 41.7 µM with 0.83% DMSO was included in the HEK293 assay in order to maximise the chances of deriving an IC_50_ value for more weakly cytotoxic compounds.

Compound activity in the retest campaign was calculated as percentage inhibition in relation to positive and negative controls, in the same manner as the primary screening campaign. The positive controls, pentamidine (2 µM final concentration) for *T.b. brucei* and puromycin (8 µM final concentration) for HEK293, were both screened in whole 384-well control plates. Whole 384-well plate controls were included after every batch of 20 compound plates, and were comprised of half a plate of negative control and half a plate of positive control. The negative control was the vehicle, 0.42 µM DMSO. The negative control was also included in column 24 of each compound assay plate to determine signal variation from plate to plate and to calculate compound activity. The exception was the 41.7 µM compound dose used in the HEK293 assay. In these plates column 24 contained 0.83% DMSO as a negative control. A separate 384-well plate containing a 13 point dose-response of the reference compounds puromycin, pentamidine and diminazene, in triplicate, was also included per 20 compound test plate batch to estimate assay sensitivity.

### Medicinal chemistry analysis of retest actives

Cluster analysis of the active compounds (n = 205) identified and confirmed from the primary and follow up retest campaign was performed using Pipeline Pilot. A predefined functional class fingerprinting set (FCFP_6, average number of molecules per cluster = 5, max distance to center = 0.6) was applied, followed by the removal of compounds carrying toxicophores (n = 35) or permanent charge (n = 25) based on filters developed in-house, which include a list of 110 undesirable chemical moieties. The remaining clusters (n = 93, total of 137 compounds) were then independently scored by 3 medicinal chemists with industrial experience. Scoring was based on criteria including activity and selectivity, number of active analogues in the cluster, drug-like structural features, chemical tractability, presence of additional toxicophores not detected by the previously applied filters, potential for CNS penetration, and possible overlap with scaffolds already considered for HAT development at DND*i*, or the literature.

### Compound resupply and quality control (QC)

Following medicinal chemistry analysis, compounds deemed to be of most interest were re-purchased or re-synthesised and analysed by liquid chromatography-mass spectrophotometry (LCMS) to confirm expected molecular weight and acceptable purity (>85%) prior to retesting of biological activity. These compounds were retested as in dose for N of three replicates, as described above for both *T.b. brucei* and HEK293.

### SAR mining: structure activity analysis of hit compounds

**Figure 1:**
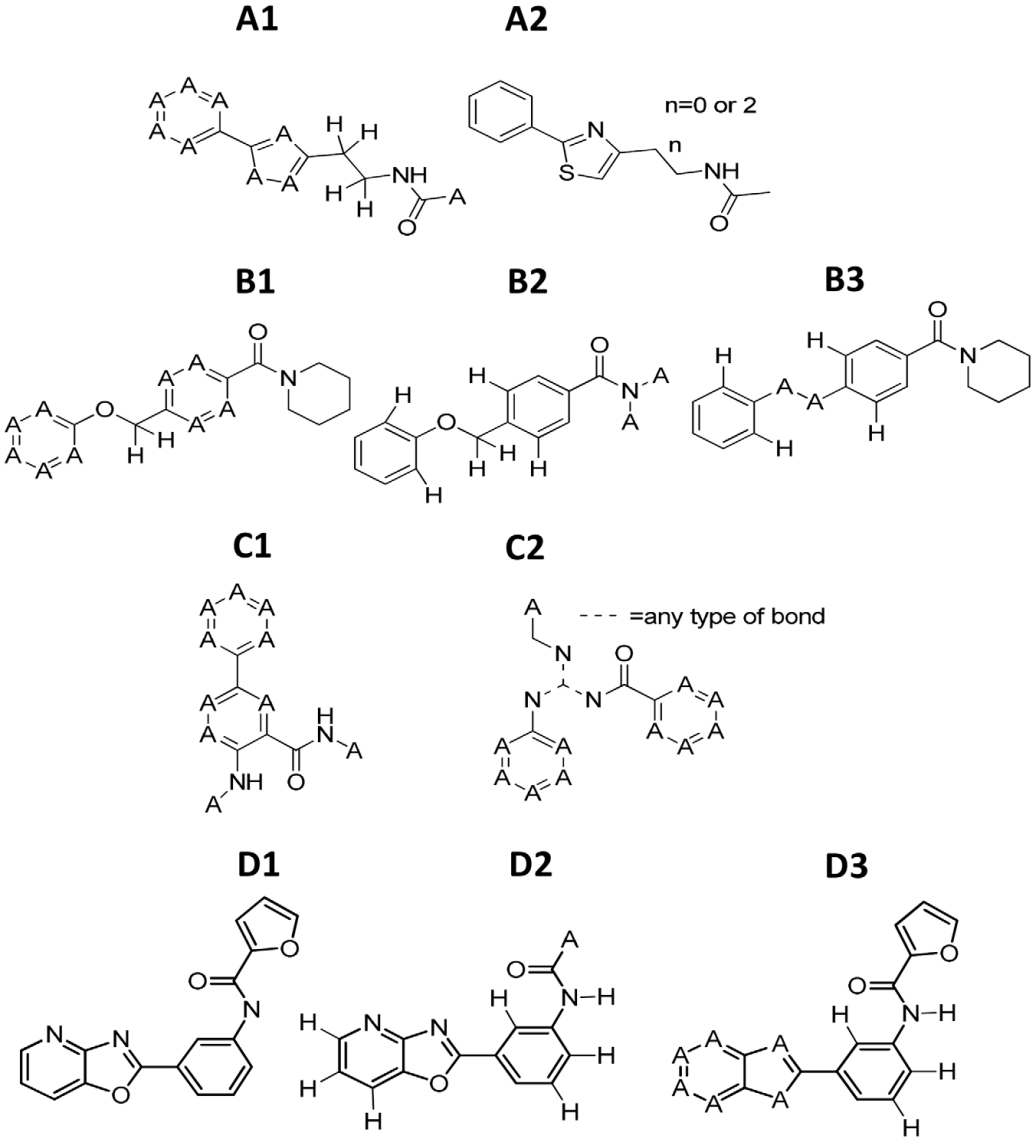
The refined substructures used for SAR mining of prioritised compounds. Parent compounds (with substructures used in the search for each) were: compounds 1–2 (A1–A2); compound 3 (B1–B3); compound 6 (D1); compound 8 (E1); compound 7 (E1–E3). In the substructures, “A” = any atom except for H. All hydrogens are made explicit.

For SAR mining, hit compounds were compared structurally to the whole primary screening compound collection. A series of substructure searches, performed in ActivityBase, were defined and refined to retrieve analogues most relevant to SAR interpretation. We have undertaken SAR mining for more than 60 HTS campaigns and have found substructure searching to return more meaningful SAR-relevant analogues than similarity searching. This is not surprising as it is well known that fingerprint-derived structural recognition captures medicinal chemistry-based structural recognition in only a rudimentary fashion [38]. The substructures that were used for searches are shown in Figure 1. A1 and A2 were the basis for searches for analogues of compounds 1 and 2, B1–B3 were used for compound 3, C1 for compound 6, D1 for compound 8, and E1–E3 for compound 7.

### Determination of the cidal action of compounds

For compounds 1, 2, 6 and 7, the minimum inhibitory concentration (MIC) was determined from a concentration response curve, generated using the Alamar Blue assay. The MIC was extrapolated as the minimum concentration at which there was a plateau of activity in the assay (>95% activity). For compound 1, this was 3.97 µM, compound 2 was 19.8 µM, compound 6 was 9.92 µM and compound 7 had a MIC of 0.99 µM. To determine cell counts at this MIC, compounds at their MIC concentration were added following incubation of 2×10^3^ parasites per well for 24 hours in the absence of compound. Cell numbers were determined after 24, 48 and 72 hours exposure to compounds and compared to controls of puromycin, also at an MIC concentration (1.15 µM). Puromycin was used as a positive control for 100% cell death (cidal action), as since for this drug there were no parasites remaining in the treated wells following 24 hours. The MIC calculated for pentamidine was 0.04 µM.

### Time to kill assay

IC_50_ values were determined for compounds 1, 2, 6 and 7 following exposure of *T.b. brucei* to each compound for 29, 48 and 72 hours. The starting dose was 40 µM, and the IC_50_ values were determined from a 16 point dose-response curve. The assay conditions were the same as previously described for the Alamar Blue assay, except that 10 µL of a final 10% concentration of Presto Blue in HMI-9 medium was added as the indicator of viable cells, at various time points. At the first time point, following 20 hours of incubation with compounds, Presto Blue was added to the wells and incubation at 37°C continued. Plates were read every hour and returned to continue incubation at 37°C. This was performed to determine at which time point there was a reproducible signal (Z' of >0.5), using puromycin as a negative control and 0.42% DMSO as a positive control. This corresponded to a 9 hour incubation, or 29 hours incubation in the presence of the compound. After 45 hours incubation, Presto Blue reagent was added, and the samples incubated for an additional 3 hours, thus read at 48 hours to give a reproducible signal. Similarly, at 70 hours, reagent was added, samples incubated for another 2 hours and read at 72 hours. If a compound reached a plateau of activity and no cells were identified at the MIC, compounds were considered to have been effectively cidal at that time point.

## Results

### Primary screening campaign

**Figure 2:**
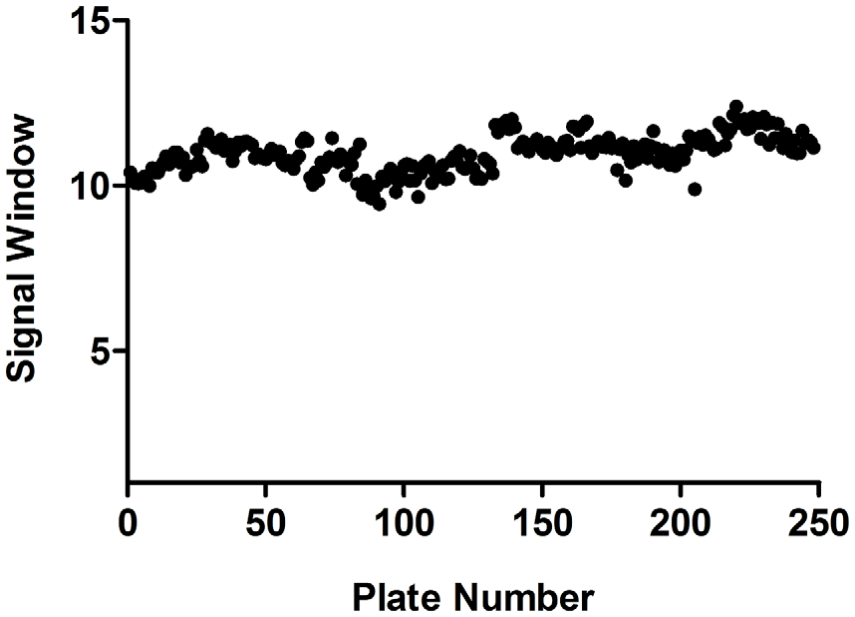
Signal window in the *T.b. brucei* primary screening campaign. The signal window for each of 248 plates containing test compounds in the primary screening campaign, expressed as a ratio of the negative to positive controls. Each dot represents the signal window calculated for a single plate. Control plates, one per 20 test compound plates, contained half a 384-well plate of 2 µM of the positive control compound, pentamidine. Negative assay controls, of 0.42% DMSO, were contained in column 24 of each assay plate containing test compounds, or 16 wells in total. The signal window was based on the average of column 24, divided by the average of the positive control taken from the control plate.

**Figure 3:**
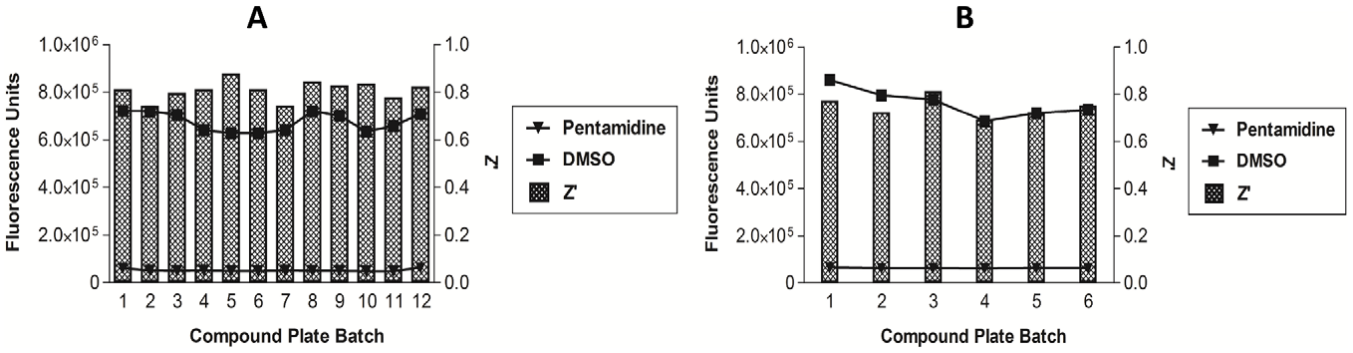
Signal and reproducibility of negative and positive controls in the *T.b. brucei* primary and retest assays. The negative and positive control assay signals (fluorescence intensity, left Y-axis) taken from whole control plates (one per 20 test compound plates) in the primary screening (A) and retest (B) screening campaigns. Negative controls were averaged from wells containing 0.42% DMSO and positive controls were from wells containing 2 µM pentamidine. The bar plots show the Z'-factor (Z', right Y-axis) for each control plate, a measure of the reproducibility of the controls in the assay.

**Figure 4:**
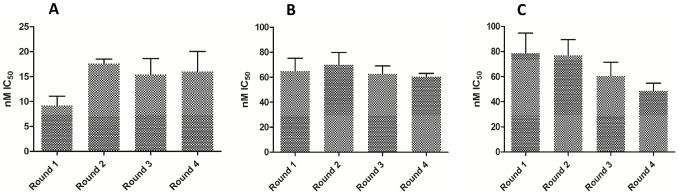
Reference compound activity against *T.b. brucei* in the primary screening campaign. Dose-response curves for the reference compounds pentamidine, puromycin and diminazene against *T.b. brucei* in the primary screening campaign. Means and standard deviations of replicate IC_50_ values were (A) pentamidine, 14.7±4.7 nM (B) puromycin, 61.86±6.8 nM and (C) diminazene, 65.4 nM±12.5 nM. Data is representative of 13 control plates each containing 3 replicates of the compounds in dose, with batches indicative of daily screens.

As a hit threshold, three times the standard deviation plus the mean of the activity of the compound collection was calculated at 50%, in an effort to reduce false positives in the assay. Compounds with ≥50% activity were therefore considered active. From the primary screening campaign, 1,980 compounds inhibited *T.b. brucei* growth by ≥50%, a hit rate of 2.27%. These were grouped into two classifications, the first containing those compounds that inhibited growth between ≥50% and <80% and the second consisted of compounds with inhibitory activity of ≥80%. Group two was comprised of 1,217 compounds and it was these that were progressed to initial retesting. In-plate controls revealed little variation in the assay signal expressed as a ratio of maximum signal to background, throughout the entire test period (Figure 2). From separate whole plate controls, the Z' was calculated as an average of 0.81±0.05 (Figure 3). IC_50_ values for each of the reference compounds determined over the four screening days are shown in Figure 4. For each screening day, there were four control plates containing a dose-response of each reference compound, in triplicate, starting at doses of: puromycin 120 µM, pentamidine 70 µM and diminazene 80 µM. One control plate was included for screening per 20 compound plate batch. Thus, there were 4 control plates each in the first 3 days of screening (80 library compound plates per day) and only 1 control plate for the last (8 library compound plates). Mean IC_50_ values and standard deviations were therefore calculated from 12 replicates each on days 1–3, and 3 replicates of dose-response of reference compounds on day 4. These values were not significantly different from one another, as determined by a one way ANOVA in GraphPad Prism, with a significant difference of P<0.05. An IC_50_ value was not considered reproducible if varying more than 3 times from the mean. All values fell within three times the mean. IC_50_ values for the reference compounds were 61.9±6.8 nM for puromycin, 65.4 nM±12.5 nM for diminazene and 14.7±4.7 nM for pentamidine.

### Retest screening campaign

Of the 1,217 primary actives that were retested from stock solutions in duplicate with dose-response curves, 822 compounds (67.5%) reconfirmed in duplicate to be ≥50% inhibitory in the *T.b. brucei* assay at the serial dilution concentration point of 10.4 µM (closest to the primary screening concentration of 10.2 µM). A dose-response plateau is necessary for IC_50_ values to be determined for these compounds. Hence, a compound needed to display ≥80% inhibitory at both 41.7 µM and 20.8 µM in duplicate (although one singleton was allowed to extend to ≥70%). There were 57.6% of the 1,217 compounds that passed these criteria. For all these compounds, titration data were imported into GraphPad Prism and the IC_50_ values estimated.

Similarly for the HEK293 assay, only data whereby an IC_50_ value could be estimated were imported in to GraphPad Prism. There were 700 compounds that displayed ≥80% inhibition at both 41.7 µM and 20.8 µM in duplicate (although one singleton was allowed to extend to ≥70%). As before, criteria included a plateau of activity necessary for the calculation of an IC_50_ value. The HEK293 IC_50_ value could be estimated for 10% of the 700 compounds in this manner and this allowed for the determination of the SI. For the remainder of compounds, an estimation of the IC_50_ against HEK293 cells was possible by observing the lowest concentration in the HEK293 assay that displayed ≥50% inhibition in at least one of the two replicates.

Using these analyses, there were 205 (29%) of the 700 re-confirmed compounds that had an estimated SI of ≥10. Of these compounds, 8 produced a non-sigmoidal curve in the *T.b. brucei* assay and therefore could not have an IC_50_ value, nor SI estimated. This may have been due to compound solubility, or the nature of the compound's action, and these compounds were de-prioritised. This left 197 hits that were progressed to medicinal chemistry cluster analysis.

Control plates, used as a measure of reproducibility, showed that the *T.b. brucei* assay had an average Z' of 0.74 (Figure 3). For the HEK293 assay, the mean Z' was 0.73 for both 0.42% DMSO and 0.83% DMSO final assay concentrations. Puromycin was active on both cell lines with an IC_50_ of 138.5±15.7 nM against *T.b. brucei* and 1123±155 nM against HEK293. Puromycin, a known cytotoxic compound therefore exhibited an SI of less than 10, supporting the use of the Alamar Blue for the identification of cytotoxic compounds. For *T.b. brucei*, diminazene exhibited an IC_50_ value of 29.5±5.8 nM and pentamidine 7.8±3.6 nM. Neither pentamidine nor diminazene displayed activity in the HEK293 assay at the doses screened (1 µM and 40 µM, respectively).

### Medicinal chemistry analysis of retest actives

Scoring was attributed independently by 3 medicinal chemists with industrial experience and was based on criteria including criterion 1: activity and selectivity (compounds with IC_50_ values indicating good activity and high selectivity were favored); criterion 2: number of active analogues in the cluster (cluster with n>1 were preferred to singletons), criterion 3: drug-like structural features (based on Lipinski's rule of 5 scoring [39]), criterion 4: chemical tractability (based on personal experience, as well as availability of commercial analogs), criterion 5: presence of additional toxicophores not detected by the previously applied filters (personal experience), criterion 6: potential for CNS penetration such as molecules with low PSA, low molecular weight, low clogP and low number of H-bond donor/acceptors [40]. It is recognised that this method is internally consistent, however may differ from analysis undertaken by other medicinal chemists [41]. This analysis lead to the selection of 11 compounds for retesting.

### Re-synthesis and rescreening of compounds

**Figure 5:**
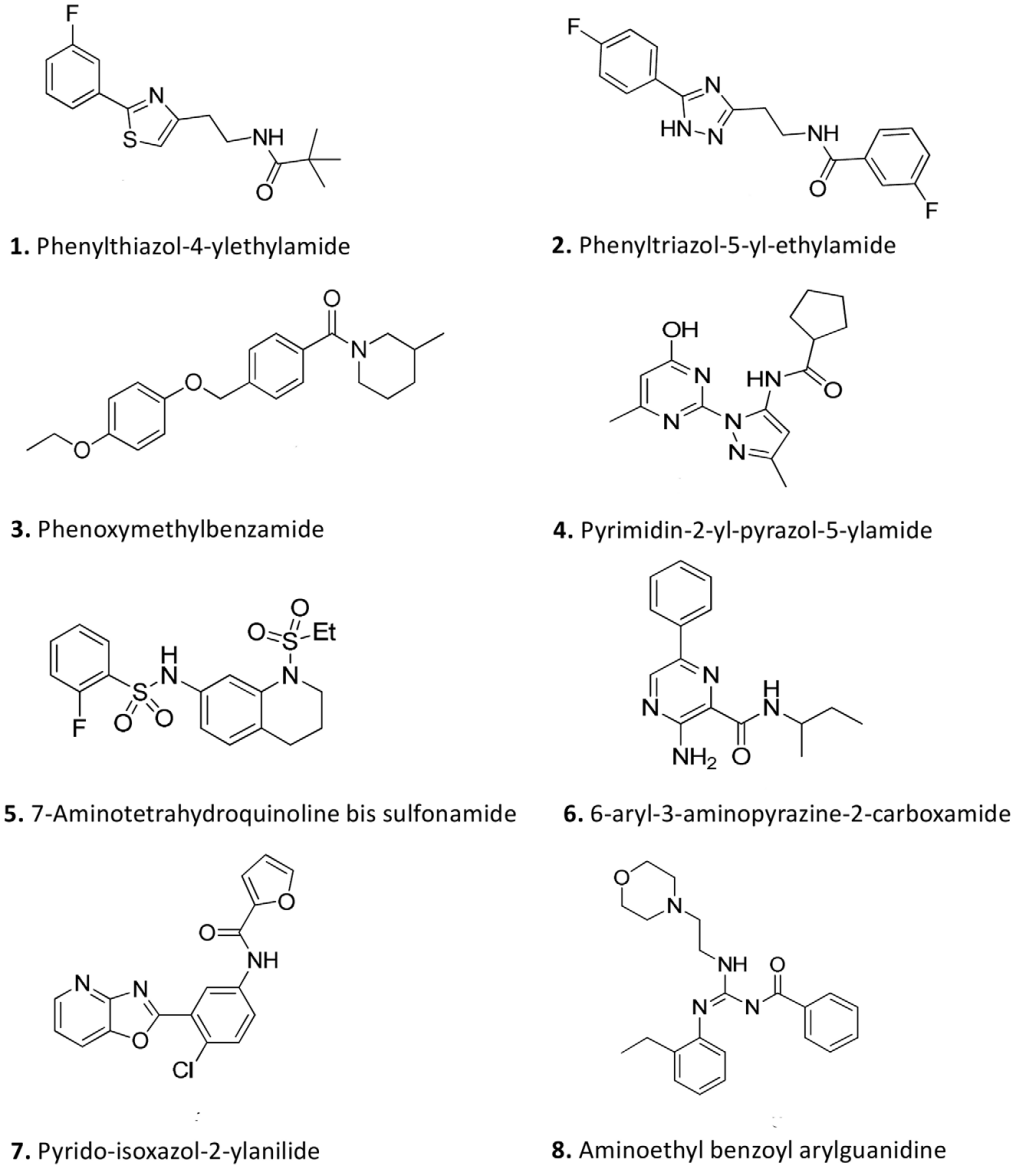
Priority hit compounds with activity against *T.b. brucei* and *T.b. rhodesiense*. Compound class and compound structures of the 6 priority hits identified from the *T.b.brucei* screening campaign, following screening against other protozoal species, medicinal chemistry analysis and consideration of optimal physiochemical properties.

The 11 compounds identified from medicinal chemistry analysis were either re-synthesised or re-purchased, and re-tested in both the *T.b. brucei* and HEK293 assays. Following this, the number was reduced to 8 (Table 1) after two resupplied compounds did not confirm activity in the *T.b. brucei* assay (<50% activity, results not shown). A third compound was found to only be >50% active at the top dose of 65 µM and therefore was unsuitable for IC_50_ or SI calculation. The IC_50_ values and calculated selectivity indices of the remaining 8 compounds are outlined in Table 1, and the structures in Figure 5. During rescreening of resynthesized compounds the Z' for the *T.b. brucei* assay was 0.81±0.02 and 0.88±0.01 for the HEK293 assay. In the *T.b. brucei* assay, pentamidine displayed an IC_50_ value of 3.52±0.36 nM, diminazene 121±9.04 nM and puromycin 58.4±0.77 nM (Table 1). In the HEK293 assay, puromycin was active at 518±28.1 nM, whilst as expected neither pentamidine nor diminazene displayed activity at the doses screened (1 µM and 40 µM, respectively). The selectivity index for puromycin was similar to that found at original retest, (8.9 fold, Table 1), as expected for a non-selective inhibitor.

**Table 1:** Activity of re-isolated compounds identified from the *T.b. brucei* primary and retest campaigns.

	Compound activity (µM)					Selectivity Index				
Compound^1^	*T.B* (Hill Slope)	*T.R*	*L.D*	*P.F*	*T.C*	*T.B* 2	*T.R* 3	*L.D* 3	*P.F* 3	*T.C* 3
1	0.785±0.0829(3.89)	1.47±0.390	46.1±22.4	35.4±2.75	2.32±0.0601	>96.0	42.3	1.35	1.76	26.8
2	4.00±0.0555(17.0)	6.77±0.545	47.3±0.324	23.9±8.48	19.3±0.0905	>19.0	17.6	2.53	5.00	6.20
3	1.13±0.347(3.21)	0.854±0.332	16.1±1.70	14.3±6.64	19.1±5.32	>67.1	58.5	3.09	3.48	2.56
4	3.08±0.290(3.33)	4.85±2.08	0.488±0.154	8.17±4.00	15.4±4.29	24.6	0.186	1.84	0.110	0.0588
5	2.11±0.130(3.09)	14.4±0.0995	6.60±1.56	12.62±5.87	14.76±0.151	>35.9	1.51	3.29	1.72	1.47
6	1.16±0.304(0.785)	0.967±0.450	34.0±5.60	11.33±4.79	19.1±5.32	>65.3	18.4	0.525	1.58	0.933
7	0.218±0.00714(6.80)	0.586±0.073	1.82±0.277	7.76±3.22	0.230±0.0496	>344	38.4	12.5	2.92	98.6
8	2.61±0.320(3.03)	0.875±0.065	>263	5.33±2.04	82.1±1.69	>29.0	150	NA	24.8	1.61
Reference Compound	Reference Compound Activity (nM)									
Puromycin	58.4±0.769(4.90)					8.90				
Pentamidine	3.52±0.360(1.65)					>283				
Diminazene	121±9.04(4.25)					>330				
Melarsoprol		6.28±1.78					2915			
Miltefosine			365±93.7					392		
Chloroquine				164±24.7					507	
Benznidazole					1680±1930					>206

Species names are abbreviated. T.B = *T.b. brucei*; T.R = *T.b rhodesiense*; L.D = *L. donovani*; P.F = *P. falciparum*; T.C = *T. cruzi*.

NA is not applicable as the IC_50_ value could not be determined within the dose range.

(1) Compound numbers refer to those outlined in Figure 4.

(2) SI was calculated with relative IC_50_ values of *T.b. brucei* and HEK293 cells. Value is described as “>” if the compound exhibited less than 50% activity at the top dose screened in the HEK293 assay at 75.8 µM, therefore an IC_50_ could not be estimated.

(3) SI was calculated with relative IC_50_ values of *T.b. rhodesiense*, *L. donovani*, *P. falciparum* and *T. cruzi* to L6 cells.

The standard deviation was calculated from two experiments with one replicate in each for the *T. cruzi*, *L. donovani* and *P. falciparum* assays and from three experiments containing two replicates each for the *T.b. brucei* assay.

Compound activity against *T. b. brucei* and a panel of human infective protozoal species, including the HAT species *T.b. rhodesiense* of compounds identified from the *T. b. brucei* screening campaign. These compounds had the most favourable overall biological and medicinal chemistry profiles.

### Screening against related protozoal species

The compounds identified by medicinal chemistry analysis as the most promising were also tested in dose-response against the human infective parasites *T.b. rhodesiense*, *L. donovani* and *T. cruzi* to estimate IC_50_ values. Data obtained is shown in Table 1. Rat skeletal L6 muscle cells were also used as an indicator of cytotoxicity and the SI was calculated against all species. Initial analysis of compound activity was made against the HAT reference strain, *T.b. rhodesiense*, taking into consideration the IC_50_ value and the SI. Criteria used were as described for the primary screening and retest campaigns, therefore for compounds to be initially considered as favourable hits for further progression, the IC_50_ cut off was <10 µM and the SI>10. Compound 5 had an IC_50_ value <10 µM and a corresponding SI of <10, and thus was de-prioritised. Compound 4 displayed an SI of 0.19 and therefore was also de-prioritised. This left a panel of 6 compounds to be considered for further progression. Table 2 shows the physiochemical properties of these 6 prioritised compounds: the molecular weight, aqueous solubility, polar surface area and cLogP.

**Table 2:** Physicochemical properties of the top 6 hit compounds.

Compound^1^	mw	Calculated aqueous solubility (µM)	Polar Surface Area (A^2^)	cLogP
1	306	63	42	3.4
2	328	8	71	2.9
3	353	25	39	4.8
6	270	3,160	81	2.0
7	339.5	0.025	81	2.6
8	380	32	67	3.1

1 Compound numbers refer to those outlined in Figure 5.

Reference compounds were used as controls throughout testing with all of these assays and are also shown in Table 1. For the *T.b. rhodesiense* assay, the drug melarsoprol displayed an IC_50_ of 6.28±1.78 nM. Benznidazole, a drug used to treat Chagas disease, was 1680±1930 nM active and miltefosine, a treatment for Leishmaniasis had an IC_50_ value of 365±93.7 nM. The drug chloroquine was active against *P. falciparum* with an IC_50_ of 164±24.7 nM.

### SAR mining: structure activity analysis of hit compounds

For the 6 hit compounds, ActivityBase was used for substructure searching to identify the relevant analogues to associate with the primary screening data. The refined substructures used for searches are shown in Figure 1. Tables S1, S2, S3 and S4 show structure and activities of these compounds over the *T.b. brucei* primary screening and retest campaigns. Identified analogues are shown in Supplementary Table S1 (compounds 1 and 2), Table S2 (compound 3), Table S3 (compound 6) and Table S4 shows analogues of compound 7. No analogues of compound 8 were found in the library using these methods, even using the relatively broad substructure definition D1 in Figure 1.

### Determination of the cidal action of compounds

**Figure 6:**
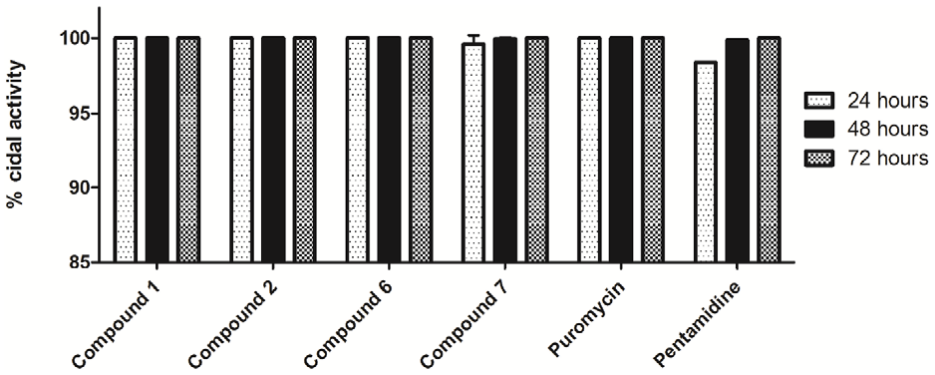
Determination of the cidal activity of compounds 1, 2, 6 and 7 over 72 hours. Death of *T.b. brucei* cells in wells estimated by parasite counts at 24, 48 and 72 hours following addition of the minimum inhibitory concentration (MIC) of each compound. The positive control was puromycin.

Measurements of the number of *T.b. brucei* cells, following exposure to the MIC of compounds 1, 2, 6 and 7 during a 72 hour period, are shown in Figure 6. Treatment with three of the 4 compounds at the MIC for 24 hours resulted in cell counts indicating the complete lack of viable trypanosomes. However, the compound pyrido-isoxazol-2-ylanilide (compound 7), only cleared parasites following 72 hours incubation. At the MIC of puromycin, no cells remained following 24 hours treatment, whereas with pentamidine this effect was not observed until 72 hours incubation at the MIC.

### Time to kill assay

The IC_50_ values for all 4 compounds selected for cidal assessment did not differ between the Presto Blue assay at 72 hours and the Alamar Blue assay (total compound exposure in this assay is also 72 hours), as shown in Table 3. Thus the Presto Blue assay was considered to also be an accurate indicator of compound activity measured over time and the results were comparable to IC_50_ values determined in the Alamar Blue assay. All compounds were active at 29 hours, with a plateau of activity displayed in dose-response curves. Compounds 1, 2 and 7 showed similar IC_50_ value across all time points, while compound 6 reached a stable IC_50_ value at 48 hours incubation with the compound (Table 3). Puromycin and pentamidine were demonstrated to reach a maximum IC_50_ value after 48 hours exposure.

**Table 3:** Time to kill estimated by the IC_50_ values of compounds 1, 2, 6 and 7 over 72 hours.

Compound^1^	Activity (nM IC_50_ value)			
	29 hours	48 hours	72 hours	AB^1^ assay
1	898±332	691±66.4	1030±18.4	1100±65.1
2	4840±449	3830±873	500±43.1	4710±272
6	3090±1380	177±11.0	111±50.2	160±40.3
7	178±7.00	142±38.7	220±17.6	198±1.28
Puromycin	152±65.4	58.1±10.1	68.6±0.283	64.1±0.240
Pentamidine	9.13±2.76	3.41±0.661	4.66±0.936	5.81±0.707

The IC_50_ values for each compound were determined at 29, 48 and 72 hours following the addition of compound by a Presto Blue-based assay, over 2 experiments. The IC_50_ was also determined for the Alamar Blue-based HTS assay format. The positive control was puromycin and the reference compounds were puromycin and pentamidine.

1 Compound numbers refer to those outlined in Figure 5.

## Discussion

Due to the many problems associated with current existing treatments for HAT, in particular toxicity, treatment regimes and cost, there exists a tangible need for new compounds to be introduced into early HAT drug discovery. HTS has been utilised by a number of research groups for HAT to identify active compounds for the drug discovery process, however there are few incorporating the use, or development of HTS for whole cells [20], [42], [43]. The inclusion of an assay to estimate cytotoxicity as a part of a whole cell HTS campaign is an important consideration for the progression of potential compounds. Here we describe the utilisation of an Alamar Blue HTS assay [20] to successfully screen a library of almost 90,000 small molecules. Following medicinal chemistry analysis of the positive hits in the assay, eight compounds with activity against *T.b. brucei* were identified. These compounds had IC_50_ values ranging from 0.22 µM to 4 µM with associated selectivity indices ranging from 19 to greater than 345.

Both the primary and retest screening campaigns were reproducible as exemplified by the statistical coefficient of the Z'. For the primary screening campaign, the Z' was averaged at 0.81 for the *T.b. brucei* assay (Figure 3). At retest the respective Z' values were 0.74 and 0.73 for *T.b. brucei* and HEK293 assays. Throughout the campaign, reference compounds in the *T.b. brucei* assay were within the range of the IC_50_ value of previously reported results for the same assay format [20]. The reproducibility of the reference compounds over primary screening days is highlighted in Figure 4. The HEK293 assay was validated in this campaign as effective for the identification of cytotoxic compounds by the activity of the compound puromycin. Puromycin is a general cell growth inhibitor of both eukaryotic and prokaryotic cells which disrupts protein synthesis. It was active on both cell lines in the retest screening campaign with an IC_50_ of 138.5±15.7 nM against *T.b. brucei* and 1123±155 nM against HEK293. This compound would therefore have been correctly identified as non-specifically cytotoxic by our criteria that a potentially useful *T.b. brucei* active must have an initial SI >10. This was also shown through the data obtained for the controls from during screening of re-isolated compounds, where the SI for puromycin was 8.9 (Table 1). As anticipated, neither pentamidine nor diminazene, which are registered drugs against HAT and *T.b. brucei*, respectively, exhibited activity in the HEK293 assay at the doses screened. Diminazene is reported to have an SI of 692 [44], whilst pentamidine has low µM activity reported for some mammalian cell lines [45].

The 8 compounds identified following reconfirmation of actives from new solids and chemical clustering were subjected to testing against the human HAT infective species *T.b. rhodesiense*, as well as other protozoal species that cause disease such as *T. cruzi* (Chagas disease), *L. donovani* (Leishmaniasis) and a chloroquine and pyrimethamine resistant strain of *P. falciparum* (Malaria). The structures and chemical classes of these compounds, designated compounds 1 to 8, are shown in Figure 5. As an additional mammalian cytotoxicity control and one relevant when screening these additional assays for protozoal parasites, the rat skeletal myoblast L6 cell line was used as this cell line is the host cell line used for the *T. cruzi* assay. The biological activities of these 8 compounds against *T.b. brucei*, a panel of human infective parasite species, plus the L6 cytoxicity data, with corresponding HEK293 selectivity indices are shown in Table 1. The activity of the relevant control/reference drugs has also been included. On the basis of this data, 2 compounds displayed relatively low (Table 1, compound 5) or extremely low (Table 1, compound 4) SI and thus were not considered favourable for progression. This left 6 high priority compounds, representing 5 distinct structural classes that could serve as a basis for progression in the early drug discovery process for HAT. Structures and key physicochemical properties for selected compounds are listed in Table 2. For analysis of physicochemical properties, a cLogP of 1–4 is considered favorable; >4–6 is acceptable, while >6 is unfavorable. A preferred solubility is considered to be >10 µM. Polar surface area is considered to be good at less than 70 Å^2^ and acceptable less than 80 Å^2^. A molecular weight lower than 400 is preferred in terms of lead-likeness and blood brain barrier crossing properties.

The phenylthiazole amide (compound 1) was active against *T.b. brucei* with an IC_50_ value of 0.79 µM and an SI of >96. It was similarly active against *T.b. rhodesiense* with an IC_50_ of 1.5 µM and an SI of 42. This compound also demonstrated activity against *T. cruzi* with an IC_50_ of 2.3 µM. In terms of physicochemical properties, it has a low molecular weight of 306, predicted good aqueous solubility of 63 µM, a low polar surface area of 42 Å^2^ suitable for CNS penetration, and a favourable cLogP of 3.4. Phenyltriazol-5-yl-ethylamide (compound 2), although closely related, was significantly less active against *T.b. brucei* with an IC_50_ value of 4.0 µM, with also a weaker *T.b. rhodesiense* activity IC_50_ of 6.8 µM. The SI for compound 2 determined against both HEK293 and L6 cells was approximately 20. The physicochemical properties of this compound reveal it to be of low molecular weight, with an acceptably low polar surface area of 71 Å^2^ and a favourable cLogP of 2.9, although the calculated aqueous solubility is low at 8 µM. A literature search revealed no biologically active compounds closely related to these two hit compounds, suggesting that these compounds may represent starting points for novel trypanocides. There were approximately 3 dozen compounds related to compound 1 (Table S1), approximately two dozen of which (1, 4–7, 16–24, 26–28, 33–36) were structurally very similar. Few of these exhibited any activity, suggesting tight SAR around the core structure. The exception to this was the potent thiophene-containing compound (entry 23), that did not initially pass the medicinal chemistry functional group filters, because of the thiophene group. However, this compound still provides useful SAR and suggests that different hydrophobic amides may be tolerated in this region with retention of potent activity. Remaining compounds were more distant, conformationally constrained, or contained heterocyclic alternatives to the thiazole and none were active.

The phenoxymethylbenzamide (compound 3) had moderate activity against *T.b. brucei* with a retest IC_50_ of 1.1 µM and an SI of >67. It was similarly active against *T.b. rhodesiense* with an IC_50_ of 0.85 µM and an SI of 60. In terms of physicochemical parameters, this compound has a moderately low molecular weight of 353, a calculated aqueous solubility of 25 µM, a low polar surface area of 39 Å^2^ and an acceptable cLogP of 4.8. SAR mining revealed 34 analogues related to compound 3 (Table S2). Some of these compounds had only relatively minor changes (entries 5, 11, 13, 33) but of these, only one (entry 13) showed some activity (77% at 10.4 µM), suggesting both ends of the molecule (piperidine amide and p-alkoxyphenyl) are likely to be important for activity. The remaining compounds tended to have more significant changes to both ends or the central unit and none of these were active except for one (entry 3), suggesting the piperidine could be replaced with a diaminoethane though cytotoxicity would need to be monitored.

The activity of the pyrimidin-2-yl-pyrazol-5-ylamide (compound 4) was 3.1 µM for *T.b. brucei*, with an SI of 25. This compound also demonstrated activity against *T.b. rhodesiense* IC_50_ of 4.8 µM but with an extremely low SI of 0.19 to L6 cells, and it was for this reason that this compound was not included in the top 6 compounds to be considered further. The 7-aminotetrahydroquinoline bis sulfonamide (compound 5) had a moderate retest *T.b. brucei* IC_50_ value of 2.1 µM and an SI of 36 to HEK293 cells. However the low activity observed against the infective species (*T.b. rhodesiense*) of 14 µM rendered this compound de-prioritised.

None of the entries 1, 2, 3 or 5 belong to classes associated with any known biological activities as far as the authors can ascertain. However, this is not the case for compound 6, 6-aryl-3-aminopyrazine-2-carboxamide, which was moderately active with a retest IC_50_ of 1.2 µM and an SI of >65 when cytotoxicity is measured on HEK293 cells. It was similarly active against *T.b. rhodesiense* with an IC_50_ of 0.97 µM and an SI to L6 cells of 18. This compound is predicted to have a favourable aqueous solubility of 3.2 mM, has a low molecular weight of 270, an acceptably low polar surface area of 81 Å^2^ and a favorable cLogP of 2.0. This class is quite heavily patented and associated with numerous biological activities [46]–[50]. Only one compound was a close analogue of compound 6, a des-N-alkyl carboxamide (Table S3), however this was inactive, suggesting the alkyl group is essential for activity.

The pyrido-isooxazol-2-ylanilide (compound 7) is an isoxazol-2-ylanilide with a fused pyridine ring and displays the best biological activity profile of all compounds, with a *T.b. brucei* retest IC_50_ value of 0.22 µM and an SI of >345. It was similarly active against *T.b. rhodesiense* with an IC_50_ of 0.59 µM and an SI of 39. This compound also displayed activity against *T. cruzi* with an IC_50_ of 0.23 µM and an IC_50_ against *L. donovani* of 1.8 µM, suggesting potential as a broad spectrum anti-kinetoplastid. The physicochemical properties of this compound are favourable, with a moderately low molecular weight, an acceptable polar surface area of 81 Å^2^ and a favourable cLogP of 2.6. The calculated aqueous solubility is low (25 nM) and it is possible the actual solubility may be improved due to the ortho effect of the 2-chloro substituent. This compound belongs to a class with an isolated report of biological activity, activation of the NAD+-dependent deacetylase SIRT1 [51], a sirtuin, which also appears to be present and important in trypanosomes [52]–[54]. This compound would appear to present a promising starting point for drug development, though early investigation of aqueous solubility and its improvement could be important. For this compound, there were 19 analogues that provided useful SAR (Table S4). Several compounds suggested the furan was important for activity, as replacement with substituted phenyl ring (entries 6, 10, 11, 16, 17, 19) or extension (entries 5, 7, 9, 12, 15, 18) led to inactive compounds. However, replacement with a simple propyl group (2) led to an active compound suggesting smaller hydrophobics may be acceptable. Two compounds had small changes in other parts of the molecule and were also inactive, suggesting even simple substitution changes to the central phenyl ring (entry 3) are not necessarily tolerated nor small changes to the distal pyridine ring (entry 4). While more significant in their alterations, all other analogues are still clearly related to the parent compound yet inactive, suggesting tight SAR.

The aminoethyl benzoyl arylguanidine (compound 8) displayed a *T.b. brucei* retest IC_50_ value of 2.6 µM and an SI of >29. This compound displayed increased activity against *T.b. rhodesiense* with an IC_50_ value of 0.88 µM and an SI of 150, whereas the activity against *T. cruzi* was low (IC_50_ of 82 µM). The biologically active conformation of this molecule may adopt an intra-molecular hydrogen-bonded form as shown [55], similar to benzoylureas [56]. In terms of physicochemical properties, this compound has a moderate molecular weight of 380, a reasonable aqueous solubility of 32 µM, an acceptably low polar surface area of 67 Å^2^ and a favorable cLogP of 3.1. Closely related compounds are patented as inhibitors of human mitochondrial F1Fo- ATPase [57], the same molecular target that DB289 has been suggested to target in *T. brucei* [58]. Oligomycin A, which is known to inhibit mitochondrial membrane associated ATPases in mammalian cells [59] has also demonstrated potent activity against *T.b. brucei* [60]. Oligomycin sensitive ATPases have been found to be present in *T.b. brucei* [61]. The aminoethyl benzoyl arylguanidine represents a highly tractable and attractive structure for medicinal chemistry optimization, although consideration will need to be given to the potential for liver toxicity manifested in DB289, and how this may be overcome [9]. Data mining showed there were no analogues of this compound in the library screened.

From the hit chemical classes, compounds 1, 2, 6 and 7 underwent further biological profiling to ascertain whether their action was cidal or static at the MIC determined. Of the 4 compounds profiled, all had completely cleared parasites in wells by 72 hours incubation at the MIC (Figure 6) and were therefore considered to have a cidal action. Compounds 1, 2 and 6 and the control puromycin resulted in complete depletion of trypanosomes at this dose at 24 hours, whilst compound 7 and the control compound, pentamidine, required a 72 hour incubation to attain the same effect.

To determine the IC_50_ values of compounds 1, 2, 6 and 7 over time, as an estimation of the kill time, the resazurin-based reagent, Presto Blue, was used. In the presence of live cells this dye converts more rapidly to a fluorescent end product, in comparison to Alamar Blue (results not shown). Dose-response curves of these compounds showed a plateau of activity of the 4 compounds at 24 hours (considered as 2 doses or more at >90%), suggesting that all compounds were active ≤29 hours. Compounds 1, 2 and 6 at MIC resulted in complete clearance of all parasites at ≤29 hours, with compounds 1 and 2 displaying the fastest cidal activity, with a maximum IC_50_ value reached at this point (Table 3). Compound 7 had similar IC_50_ values over each time interval investigated however at the MIC not all parasites were cleared until 72 hours. Although the MIC would shift slightly over time, at 24 and 48 hours there were 0.41% and 0.06% of the population remaining, respectively. Additional profiling revealed these compounds were cidal in nature and the speed of action was either similar to, or faster than, the known drug, pentamidine. These compounds will be profiled at reduced exposure times to determine if the time to kill may be less than the exposure times studied here. Estimation of the MIC at each time point would clarify complete parasite clearance.

Collation of all of the analyses completed led to the selection of five priority classes: phenylthiazol-4-ylethylamide, phenoxymethylbenzamide, 6-aryl-3-aminopyrazine-2-carboxamide, pyrido-isoxazol-2-ylanilide and aminoethyl benzoylarylguanidine. In summary, these compounds are novel scaffolds for HAT early drug development and represent attractive templates for further biological analysis and medicinal chemistry optimization, to build structure-activity relationships for compounds active against *T.b. brucei*. Upon confirmation of SAR, the chemistry program would be extended to optimize potency and solubility, in conjunction with early *in vitro* absorption, distribution, metabolism, elimination (ADME) and toxicity assays. Early pharmacokinetic studies (PK), to measure of brain compound levels, as well as *in vivo* efficacy studies in HAT murine models, would follow upon identification of suitable candidates. Medicinal chemistry efforts are being actively pursued to synthesize new compounds from the starting points discussed here, in a bid to generate leads with improved physicochemical and biological properties. Chemical structures and biological activities of all compounds defined as actives in the *T.b. brucei* primary screening campaign at ≥80% activity (1217), which were retested in dose-response in both the *T. b. brucei* assay and the HEK293 cytotoxicity assay are available in the CHEMBL-NTD database https://www.ebi.ac.uk/chemblntd.

## Supporting Information

Table S1 **Tables S1, S2, S3 and S4:** Analogues of the top 6 hit compounds. SAR mining revealed those compounds in the library that was screened that were structurally related to the 6 hit compounds. Tables show structure and activities of these compounds over the *T.b. brucei* primary screening and retest campaigns. Table S1 = compounds 1 and 2; Table S2 = compound 3; Table S3 = compound 6, table S4 = compound 7. SAR mining revealed no analogues of compound 8 to be present in the library.
(XLSX)

Table S2 (XLSX)

Table S3 (XLSX)

Table S4 (XLSX)
