# Correction: Identification of Compounds with Anti-Proliferative Activity against *Trypanosoma brucei brucei* Strain 427 by a Whole Cell Viability Based HTS Campaign

**DOI:** 10.1371/annotation/cc952155-c929-4e4f-8fc0-43cb84c50452

**Published:** 2013-01-22

**Authors:** Melissa L. Sykes, Jonathan B. Baell, Marcel Kaiser, Eric Chatelain, Sarah R. Moawad, Danny Ganame, Jean-Robert Ioset, Vicky M. Avery

There are various errors contained in the references 6, 7, 8, 9, 10, 25, 28, 29, 39, 40 and 43. A summary of issues is as follows:

* References 6 - 10 contain incorrect information and numbering. They should read as follows:

6. (1998) Control and surveillance of African trypanosomiasis. Report of a WHO Expert Committee. World Health Organ Tech Rep Ser 881: I-VI, 1-114.

7. Chappuis F, Udayraj N, Stietenroth K, Meussen A, Bovier PA (2005) Eflornithine is safer than melarsoprol for the treatment of second-stage Trypanosoma brucei gambiense human African trypanosomiasis. Clin Infect Dis 41: 748-751.

8. Kennedy PG (2008) The continuing problem of human African trypanosomiasis (sleeping sickness). Ann Neurol 64: 116-126.

9. Pepin J, Milord F (1994) The treatment of human African trypanosomiasis. Adv Parasitol 33: 1-47.

10. Schmid C, Richer M, Bilenge CM, Josenando T, Chappuis F, et al. (2005) Effectiveness of a 10-day melarsoprol schedule for the treatment of late-stage human African trypanosomiasis: confirmation from a multinational study (IMPAMEL II). J Infect Dis 191: 1922-1931.

* Reference 25, 28 and 29 should be removed.

* Reference 39 is incorrect and should read as follows:

Lipinski CA (2004) Lead- and drug-like compounds: the rule-of-five revolution. Drug Discov Today: Technologies 4: 337-341.

* Reference 40 is incorrect and should read as follows:

Wager T, Verhoest P, Villalobos A (2010) Moving beyond Rules: The Development of a Central Nervous System Multiparameter Optimization (CNS MPO) Approach To Enable Alignment of Druglike Properties. ACS Chem Neurosci 1.

* Reference 43 is incorrect and should read as follows:

Sykes ML, Avery VM (2009) A luciferase based viability assay for ATP detection in 384-well format for high throughput whole cell screening of Trypanosoma brucei brucei bloodstream form strain 427. Parasit Vectors 2: 54. 

